# Pattern of food allergy in adults

**DOI:** 10.1186/2045-7022-1-S1-S5

**Published:** 2011-08-12

**Authors:** André Knulst

**Affiliations:** 1 University Medical Centre, Dept. Dermatology/Allergology, Utrecht, Netherlands

## 

The pattern of food allergy in adults is different from that of children in various aspects. The typical foods involved in infants are milk, egg, peanut and probably tree nuts, whereas in older children and adults fruits and nuts are the most frequent allergenic foods. In addition, allergy to vegetables can occur. Allergy to milk and egg largely disappears at young age, but can still be present in adults, in part because of persistence from childhood, but also as de novo allergies developing in adolescents or adults.

The high prevalence of fruit and nut allergy in adults and older children is for a large part due to the pollen-food syndrome: the result of cross-reactivity between allergens in tree (birch) pollen and in fruits, nuts and vegetables. The list of foods cross-reacting with birch pollen is steadily increasing and contains members of different plant food families. Also some less frequently eaten tropical fruits are on the list. In general reactions due to the pollen-food syndrome tend to be relatively mild, but (more) severe reactions have been reported especially for soy, celery and tropical fruits. Severe reactions on first exposure of cross-reactive foods were reported.

Another important cross-reactive allergy is that between latex and foods and vice versa. The most frequently involved foods are kiwi, banana, avocado, chestnut and buckwheat.

In clinical practice the most important problem is how to confirm which are the culprit foods and how to judge the severity of the food allergy.

Studies from different geographical regions have show that there can be significant differences in the prevalence and severity of specific food allergies due a.o. to differences in pollen exposure and eating habits, which is also partly reflected in differences in patterns of allergen profiles.

The increasing knowledge on relevant allergen recognition patterns, known as component resolved diagnostics, will help to improve our insight in the relevant allergens involved, the routes of sensitisation and, at least partially, to estimate the risk of severe reactions.

